# Prediction of Pedestrian Crossing Behavior Based on Surveillance Video

**DOI:** 10.3390/s22041467

**Published:** 2022-02-14

**Authors:** Xiao Zhou, Hongyu Ren, Tingting Zhang, Xingang Mou, Yi He, Ching-Yao Chan

**Affiliations:** 1 School of Mechanical and Electronic Engineering, Wuhan University of Technology, Wuhan 430070, China; zhouxiao@whut.edu.cn (X.Z.); a453746329@163.com (H.R.); effyzh@hotmail.com (T.Z.); mouxingang@163.com (X.M.); 2 Intelligent Transport Systems Research Center, Wuhan University of Technology, Wuhan 430063, China; 3 California Partners for Advanced Transportation Technology (PATH), University of California, Berkeley, Richmond, CA 94720-5800, USA; cychan@berkeley.edu

**Keywords:** traffic safety, autonomous driving, surveillance video, behavior prediction, multi-source feature fusion

## Abstract

Prediction of pedestrian crossing behavior is an important issue faced by the realization of autonomous driving. The current research on pedestrian crossing behavior prediction is mainly based on vehicle camera. However, the sight line of vehicle camera may be blocked by other vehicles or the road environment, making it difficult to obtain key information in the scene. Pedestrian crossing behavior prediction based on surveillance video can be used in key road sections or accident-prone areas to provide supplementary information for vehicle decision-making, thereby reducing the risk of accidents. To this end, we propose a pedestrian crossing behavior prediction network for surveillance video. The network integrates pedestrian posture, local context and global context features through a new cross-stacked gated recurrence unit (GRU) structure to achieve accurate prediction of pedestrian crossing behavior. Applied onto the surveillance video dataset from the University of California, Berkeley to predict the pedestrian crossing behavior, our model achieves the best results regarding accuracy, F1 parameter, etc. In addition, we conducted experiments to study the effects of time to prediction and pedestrian speed on the prediction accuracy. This paper proves the feasibility of pedestrian crossing behavior prediction based on surveillance video. It provides a reference for the application of edge computing in the safety guarantee of automatic driving.

## 1. Introduction

Pedestrians are one of the main participants in the transportation system in urban environments. They become the most defenseless road users due to the lack of protection measures and vulnerable to the threats to life from traffic accidents. Therefore, to ensure the safe operation of autonomous vehicles, automated systems require the ability to predict pedestrian behaviors, especially at the point of crossing. Knowing as soon as possible if a detected pedestrian has the intention of intersecting the ego-vehicle path (expecting the vehicle slowing down or braking) is essential for performing safe and comfortable maneuvers preventing a crash, as well as having vehicles showing a more respectful behavior with pedestrians [1].

In the behavioral science literature, the Theory of Planned Behavior (TPB) asserts that behavior extends from intent, which in turn is a product of social-psychological attitudes, subjective norms, and perceived behavioral control. The later integrated model simplified these parts into a path, “attitude/perception-behaviors” [2]. Pedestrian crossing behavior is affected by multiple factors, including road vehicles, surrounding pedestrians, crossing intentions, and current movement speed. With the development of computer vision, image-based pedestrian behavior prediction has been widely studied [3]. Early studies mostly used single-frame picture as input into convolutional neural network (CNN) for prediction [4]. This method ignores temporal information, which plays a key role in behavior prediction. Later studies considered both spatial and temporal features, using recurrent neural networks (RNN) and three-dimensional convolution neural network (3DCNN) to extract spatio-temporal information [5,6,7,8,9]. At the same time, different methods are used to fuse a variety of features to predict pedestrians’ crossing behaviors, such as pedestrian bounding box, posture, vehicle speed and surrounding environment information [10,11,12,13,14].

The current research on pedestrian crossing behavior prediction is mainly based on vehicle-mounted videos. Due to the limitation of the line of sight, there are some hidden dangers in actual scenes. For example, when pedestrians are blocked by other vehicles or other road elements, it is difficult for the vehicle-mounted camera to observe the pedestrians who will be crossing the street. Therefore, the vehicle cannot timely detect the pedestrian crossing behavior. This is also a common cause of pedestrian crashes. With the development of Cooperative Vehicle Infrastructure System (CVIS), it is possible to use information obtained from surveillance video for autonomous driving. Internet of vehicles communication technology (V2X) makes future vehicle driving no longer rely on vehicle perception alone, but organically connect “people, vehicles, roads, clouds” and other traffic elements [15]. Connected vehicles combine the technology of Internet of vehicles and intelligent vehicles and are also a development direction of intelligent transportation systems in the future [16]. Pedestrian crossing behavior prediction based on surveillance video can be used on key roads or accident-prone areas to provide auxiliary information for vehicle decision-making, so as to reduce accident risk.

This paper focuses on the prediction of pedestrian crossing behavior based on surveillance video. The purpose is to predict whether the target pedestrian will cross the road in a period of time from the monitoring perspective. The dataset used is from a surveillance video camera installed in the University of California, Berkeley, which records a busy street on the campus. The dataset labels the pedestrian’s pose feature points. On this basis, we label the target pedestrian’s crossing behavior to make a dataset for pedestrian crossing behavior prediction under surveillance video. Meanwhile, a novel spatio-temporal feature fusion network is proposed to accurately predict pedestrian crossing behavior from the perspective of monitoring. The network integrates pedestrian posture features, local context features and global context features. Posture features include pedestrian body posture and head posture. The local context features are scene features in a certain range around the target pedestrian, which are extracted from the local scene through a convolutional neural network. The global context is the whole picture, and the optical flow field image processed by optical flow method is fused. First, perspective transformation is used to preprocess the entire image. Then convolutional neural network is used to extract the original image and optical flow field image features, respectively. In this paper, VGG19 (Visual Geometry Group 19) [17] network is introduced to extract environmental features, and the GRU [18] with attention mechanism [19] is introduced to encode pedestrian posture and context information. Finally, the obtained information is fused and the full connection layer is used to predict the pedestrian crossing behavior. In addition, this paper also studies the influence of pedestrian speed and time to prediction (TTP) on the model’s prediction performance.

The research results of this paper prove the effectiveness of pedestrian crossing behavior prediction through surveillance video. Through the acquisition and fusion of pedestrian posture, local context and global context in the video, satisfactory prediction results are achieved. This also means that it is feasible to realize the early warning of pedestrians crossing the street in automatic driving through edge calculation. The main contributions of this work can be summarized as follows: (1) Based on the surveillance dataset of the University of California, we have annotated the pedestrian crossing behavior of the target pedestrian, and produced a dataset for the prediction of pedestrian crossing behavior based on surveillance video. The dataset contains 300 video clips of pedestrians crossing the street. (2) A new spatio-temporal feature fusion network is proposed. The algorithm predicts pedestrian crossing behavior by fusing pedestrian posture, local context and global context features. Comparing multiple baseline methods, the model achieves the best results. (3) Through experiments, the effects of TTP and pedestrian speed on the prediction accuracy of each model are studied.

## 2. Related Works

Spatial-temporal features. Since the prediction task needs to consider temporal information, most studies use continuous image sequences as the input of the prediction model, which requires the model to accurately extract temporal and spatial information. Spatio-temporal modeling can extract the visual features of each frame through 2D CNN [17] or graph convolutional network (GCN) [20], and then input these features into RNN for prediction. For example, Liu [21] used the spatio-temporal context of the scene to make predictions. Firstly, each frame is parsed into pedestrians and objects of interest. Then a spatio-temporal graph centered on the target pedestrian is constructed, where features are extracted through graph convolution. Finally, RNN is used to predict the pedestrian behavior. Ullah [22] proposed a bidirectional approach where features obtained by CNN are sent in a bidirectional LSTM [23], connecting two hidden layers from opposite directions to the same output. The output layer can then simultaneously obtain information on past and future states. Another method to extract spatio-temporal information is to use the 3DCNN [6], which replaces the convolution kernel and pooling layer in the two-dimensional convolution network with three-dimensional convolution, so that the network can accept three-dimensional input and directly extract spatio-temporal features. For example, in [7,8], Spatio-Temporal Densenet is used to directly extract the features of picture sequence through 3DCNN, and then the full connection layer is used for final prediction.

Trajectory prediction. It is a common way to predict pedestrian crossing behavior through pedestrian trajectory. For example, Lee [21] proposed an RNN codec framework of deep random inverse optimal control, which predicts the future position of pedestrians and vehicles through moving targets and scene context. Luong [24] proposed a transferable pedestrian motion prediction algorithm based on inverse reinforcement learning, which can infer the pedestrian’s intention and predict the future trajectory according to the observed trajectory. On this basis, the target collision time can be estimated to remind the vehicle to avoid. Doellinger [25] used CNN to predict average occupancy maps of walking humans even in environments where information about trajectory is not available. However, pedestrian trajectory prediction is a complex task because humans may change directions suddenly depending on objects, vehicles, human interaction, etc. [26]. In these cases, it is difficult to make accurate prediction based on the trajectory.

Posture features. Pedestrian posture features are direct expressions of pedestrian intentions, such as waving, walking, observing road conditions, etc. Therefore, many researchers use pedestrian posture features to predict pedestrian crossing behavior. For example, Fang [27] used human bone key points to predict pedestrian crossing intention. The human skeleton feature points are extracted from the target pedestrian’s bounding box, and then the feature vector representing the pedestrian’s posture is established. Finally, SVM (support vector machine) classifier is used to predict pedestrian behavior. Cadena [28] proposed a model based on two-dimensional human pose estimation and graph convolution network (GCN). The extracted pedestrian posture features are represented in the form of graph, and then the processed graph sequence is input into GCN for prediction. Wang [29] proposed a fast shallow neural network classifier to predict pedestrian behavior according to the two-dimensional posture of pedestrians. Gesnouin [30] proposed SPI-Net (Skeleton-based Pedestrian Intention network): a representation-focused multi-branch network combining features from 2D pedestrian body poses for the prediction of pedestrians’ discrete intentions. However, these methods rely solely on pedestrian posture features, and ignore the information affecting pedestrian crossing behavior in the context.

**Figure 1:**
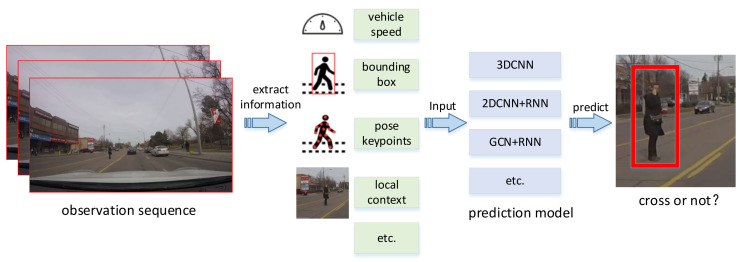
Overview of current mainstream pedestrian crossing behavior prediction methods. Firstly, the required information is extracted from the observation sequence, including pedestrian posture, pedestrian trajectory, local environment, vehicle speed and so on. Then input the information into the spatiotemporal feature processing network for behavior prediction.

Feature fusion. Some other methods focused on novel fusion architecture. Rasouli [11] proposed an architecture based on stacked RNN, which integrates five features: local context, pedestrian appearance, pedestrian posture, bounding box and vehicle speed. The features are processed hierarchically and gradually fused at each level. More complex features are input at the bottom of the model and simpler features are input at the top. In [10], a multi-modal prediction network is proposed, which uses four feature elements: global semantic map, local scene, pedestrian motion and vehicle speed. These features are gradually integrated into the network at different processing levels. In [12], a multi-task prediction framework is proposed, which takes advantage of feature sharing and multi task learning. It integrates four feature sources: semantic map, pedestrian trajectory, grid position and vehicle speed. Kotseruba [13] considered four feature sources: local environment, pedestrian posture, pedestrian bounding box and vehicle speed. A three-dimensional volume integral branch is used to encode visual information and a single RNN branch is used to process other information in parallel. Then, the attention module is introduced and applied to the hidden state of the RNN branch (temporal attention) and again to the output of the branch (modal attention). Figure 1 shows a preview of the current mainstream research on the prediction of pedestrian crossing behavior.

Compared with vehicle-mounted videos, surveillance video has a wider perspective and richer extraction of context information especially pedestrian surrounding context and vehicles on the road. The full extraction of this information can make the prediction of pedestrian crossing behavior more accurate.

## 3. Research Methodology

### 3.1. Problem Formulation

**Figure 2:**
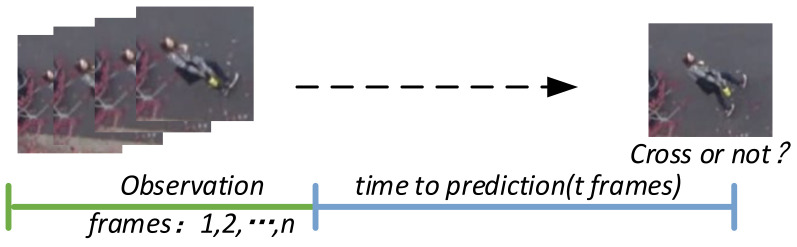
Pedestrian crossing behavior prediction task. N-frame observation is used to predict whether pedestrians cross the street after t frames.

Referring to the benchmark proposed in [5], we define pedestrian crossing behavior prediction as a binary classification problem. The goal is to predict the crossing state of pedestrian i $A_{i}^{n+t}\in \left\{0,1\right\}\;$ after t frames under the observation time of n frames, as shown in Figure 2. The prediction of the model depends on three input sources, including pedestrian posture $\left\{C_{hi},C_{bi}\right\}$, local context around pedestrians $\;C_{si}=\left\{C_{si}^{1},C_{si}^{2},\ldots ,C_{si}^{n}\right\}$, global context $\left\{C_{gi},C_{oi}\right\}$, where $C_{bi}=\left\{C_{bi}^{1},C_{bi}^{2},\ldots ,C_{bi}^{n}\right\}$ represents pedestrian body posture, $C_{hi}=\left\{C_{hi}^{1},C_{hi}^{2},\ldots ,C_{hi}^{n}\right\}$ represents pedestrian head posture, $C_{gi}=\left\{C_{gi}^{1},C_{gi}^{2},\ldots ,C_{gi}^{n}\right\}$ represents the original global context features and $C_{oi}=\left\{C_{oi}^{1},C_{oi}^{2},\ldots ,C_{oi}^{n}\right\}$ represents global optical flow field features.

### 3.2. Dataset

**Figure 3:**
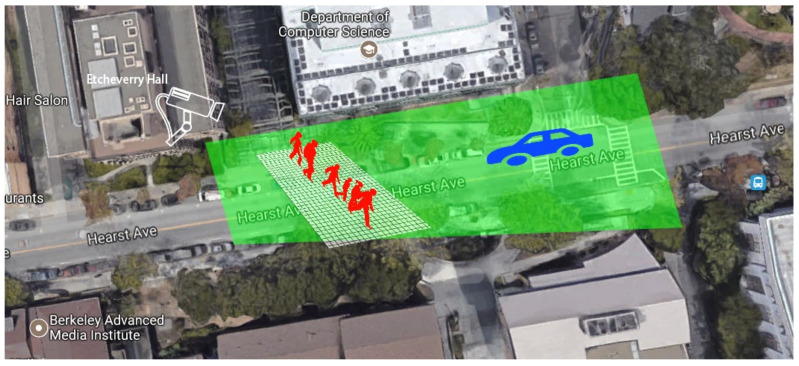
Satellite view of the experimental area. The installation position of the camera is indicated in the figure. The green area represents the camera field of view. The white speckle area is the selected area of the target pedestrian, and the target pedestrians marked in the dataset come from this area.

**Figure 4:**
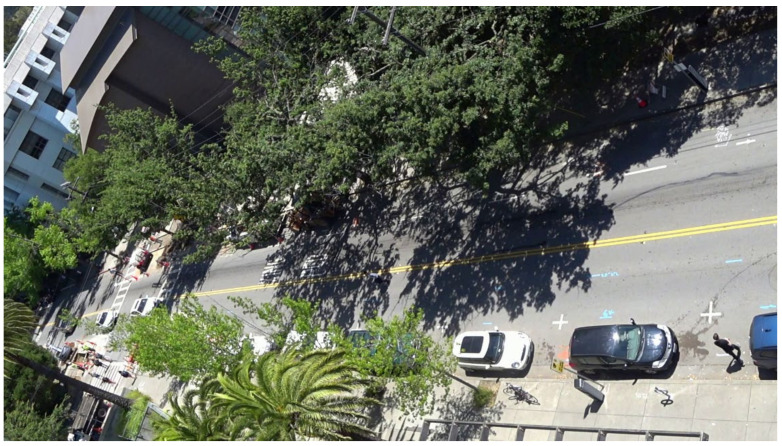
Example of monitoring scene in dataset. The camera was mounted on the top of a building with a top-down view of the street.

The dataset used in this article is recorded on a busy street on the campus of University of California, Berkeley as shown in Figure 3. The camera was mounted on the top of a building with a top-down view of the street. The field of view of the camera is shown in Figure 4.

The dataset contains 300 videos from the monitoring perspective. Each video has a target pedestrian who will cross the street. Every target pedestrian is marked when it first appears in the image boundary, and the video ends when the crossing is completed, and the pedestrian leaves the image. Each video is about 20 s long at 15 frames per second. The resolution of the video is 1920 × 1080. These videos are collected from different periods of the day. Table 1 shows the period statistics of the video data.

**Figure 5:**
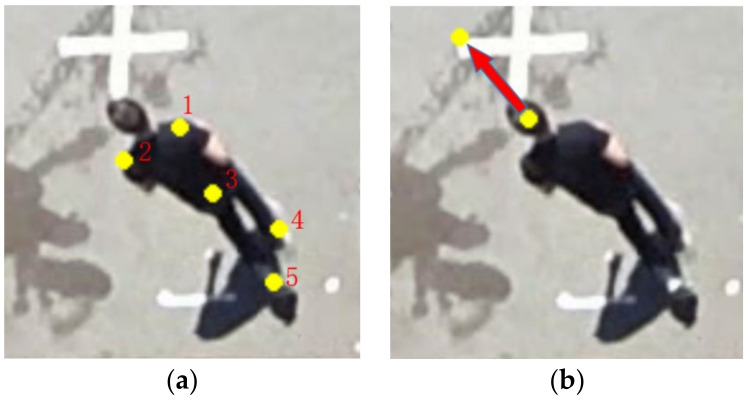
Image feature labeling: (**a**) 1–5 represent the edge of the right and left shoulders, center of the waist, right and left heels respectively; (**b**) the head position and head orientation vector.

The dataset annotates the target pedestrian’s head and body posture. The head posture is represented by two points, which represent the head position and head direction, respectively. Body posture is represented by 5 body keys. Therefore, as shown in Figure 5, the posture of each pedestrian is represented by the abscissa and ordinate of 7 points, that is, a 14D vector. On this basis, we annotate the pedestrian crossing behavior (1 represents that pedestrians are crossing the street, 0 represents that pedestrians are not crossing the street) and the number of frames where pedestrians begin to cross the street.

### 3.3. Model Construction

**Figure 6:**
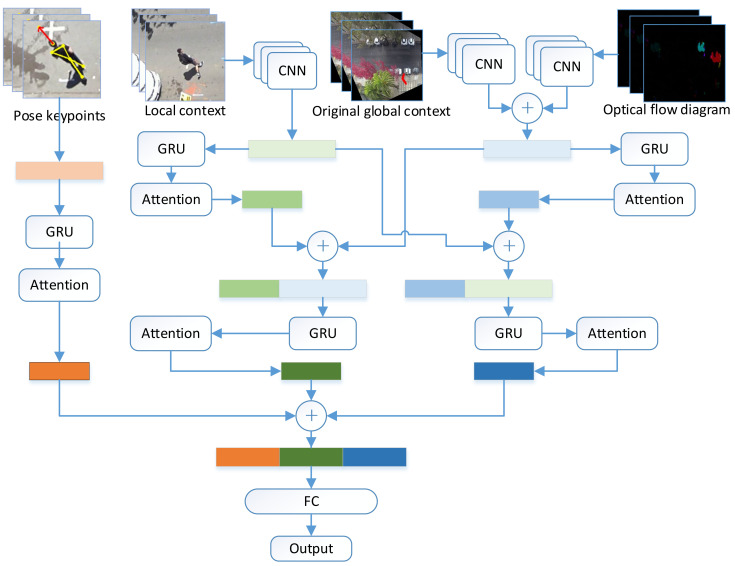
Overview of the proposed pedestrian crossing behavior prediction model. The model combines three feathers of pedestrian posture, local context and global context incorporating optical flow features. Pedestrian posture is coded by GRU, local context and global context are coded by cross-stacked GRU. Finally, the obtained features are connected and input to the fully connected layer for prediction.

We propose a new multi-source feature fusion model, as shown in Figure 6. The model integrates pedestrian pose features (body pose feature points, head pose feature points), local context and global context features. The global context features are obtained by the combination of original global context features and optical flow field features. For pedestrian pose features, we directly input them into RNN for recursive coding. Environment perception is a critical technical issue for autonomous vehicles [31]. For context features, we use cross-stacking for fusion coding of local context and global context. Firstly, CNN is used to extract the features of local scene and global context, respectively, which are put into RNN for recursion. Then, the recursive results are spliced with the other party’s features before the recursion, and the RNN is input again to calculate the deep fusion features of the environment. Finally, after stitching the vectors processed by the RNN, a 2-layer fully connected layer is used for prediction.

We use GRU [19] for recursion. Compared with the long short term memory network [23] (LSTM), GRU has a simpler structure and can achieve performance no less than LSTM on the basis of less calculation. Recalling the equation of GRU, the variables of $j{\mathrm{th}}^{}$ level of the stack is calculated as follows.

(1) $$r_{j}^{t}=\sigma \left(W_{j}^{xr}x_{j}^{t}+W_{j}^{hr}h_{j}^{t-1}\right)$$

(2) $$z_{j}^{t}=\sigma \left(W_{j}^{xz}x_{j}^{t}+W_{j}^{hz}h_{j}^{t-1}\right)$$

(3) $${\tilde{h}}_{j}^{t}=\tanh(W_{j}^{xh}x_{j}^{t}+W_{j}^{hh}\left(r_{j}^{t}⊙h_{j}^{t-1}\right))$$

(4) $$h_{j}^{t}=\left(1-z_{j}^{t}\right)⊙h_{j}^{t-1}+z_{j}^{t}⊙{\tilde{h}}_{j}^{t}$$

In the formula: $x_{j}^{t}$ represents the input at the current moment;$\;W_{j}^{xr}$, $W_{j}^{hr}$,$\;W_{j}^{xz}$,$\;W_{j}^{hz}$,$\;W_{j}^{xh}$ and $W_{j}^{hh}$ are the learnable weight matrices; $r_{j}^{t}$ and $z_{j}^{t}$ represent the reset gate and update gate weights, respectively; $h_{j}^{t-1}$ and $h_{j}^{t}$ represent the hidden layer state at the previous moment and the current moment, respectively; ${\tilde{h}}_{j}^{t}$ represents new memory at the current moment; σ is the sigmoid(·) function, and tanh(·) is the hyperbolic tangent activation function. For j=0 (the bottom level of the stack), $x_{0}^{t}$ = $c_{p}^{t}$ and for  j>0, $x_{j}^{t}=h_{j}^{t-1}+c_{p}^{t}$.

**Figure 7:**
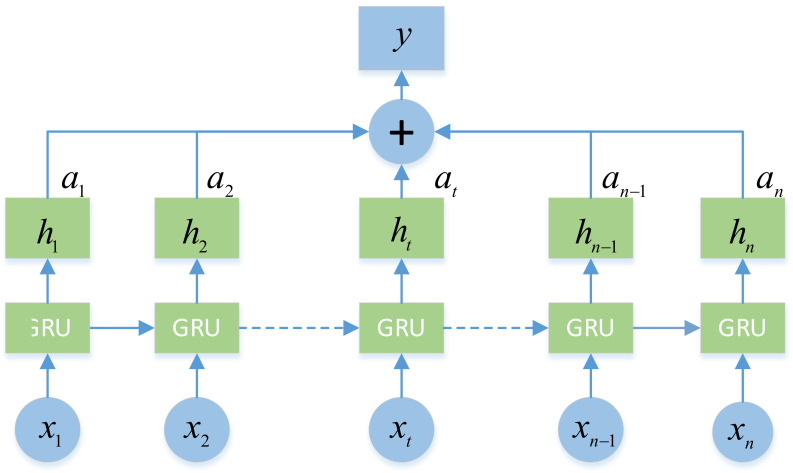
At-GRU structure, where *n* represents the input sequence length, $x_{t}$ represents the input of the *t*-th layer, $h_{t}$ represents the output of layer t, $a_{t}$ represents the weight of the timing feature calculated by the attention mechanism, and y represents the output of At-GRU, which is weighted by the output of each layer of the GRU.

Meanwhile, inspired by [3,13], we introduced the attention mechanism [18] into GRU to form At-GRU (attention-GRU). The attention module can selectively focus on some features, so as to better deal with key objects. For sequence input, the attention mechanism can assign different weights to the sequence, so as to turn the attention of the model to important features and improve the accuracy of data feature understanding without increasing the computational cost. Figure 7 shows the structure of At-GRU. Where $a_{t}$ and y are calculated as follows:

(5) $$a_{t}=\frac{\exp(\left(\tan h\left(W_{w}x_{t}+b_{w}\right){)}^{T}W_{A}\right)}{\sum_{t=1}^{n}\exp(\left(\tan h\left(W_{w}x_{t}+b_{w}\right){)}^{T}W_{A}\right)}$$

(6) $$y=\overset{n}{\underset{t=1}{\sum }}a_{t}h_{t}$$

where: $W_{w}$ and $b_{w}$ are the learnable parameters and bias of $\tanh\left(\cdot \right)$; $W_{A}$ is the learnable parameter of At-GRU.

### 3.4. Model Input Acquisition

#### 3.4.1. Pedestrian Pose Key Points

Before crossing the road, pedestrians usually walk, wave and wave their hands. In addition, the pedestrian’s head posture also reflects the pedestrian’s intention to cross the street [32]. Pedestrian posture sequence is the most direct expression of pedestrian intention. Therefore, the capture of pedestrian posture information is very important for the prediction of pedestrian crossing behavior.

The dataset used in this article has already annotated the key points of pedestrian posture. The extraction of pedestrian posture is not the focus of this article, so we directly use the ground truth pedestrian posture as the input. Pedestrian posture includes body posture and head posture. The head pose is a 4D vector, including head coordinate points and coordinate points representing the direction of the head. The vector composed of these two points can represent the direction of the head. The body posture is a 10D vector, including the horizontal and vertical coordinates of the five points of the pedestrian’s left shoulder, right shoulder, waist center, left heel, and right heel.

#### 3.4.2. Local Context

Pedestrian crossing behavior is usually affected by the surrounding context, such as zebra crossings, intersection signs, etc. In addition, when pedestrians cross the street together, the crossing intention will be greatly affected by the crossing behavior of surrounding pedestrians. Therefore, the understanding of the local context around pedestrians is helpful to predict pedestrian crossing behavior.

**Figure 8:**
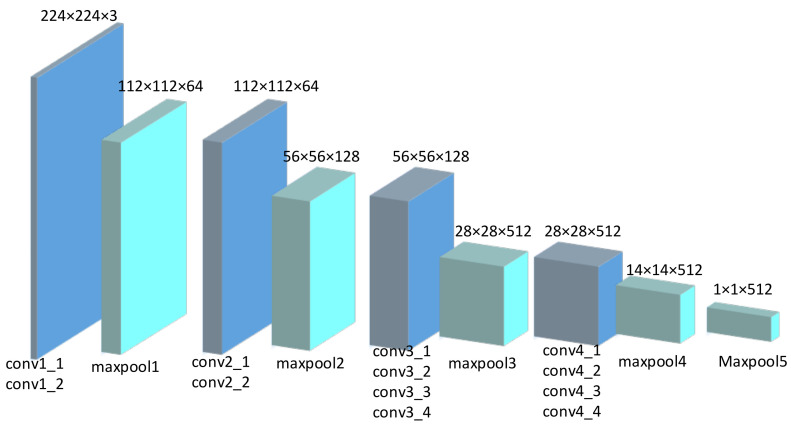
Feature extraction network based on vgg19. It extract the output from the fourth maximum pooling layer of VGG19, and then use the average pooling layer with a 14 × 14 kernel for pooling to obtain the 512D feature vector.

To define the local context, we take the waist center of the target pedestrian as the center and select the RGB image of 224 × 224 pixels around the center to form the local scene. Then we apply the pre-trained VGG19 [17] model on the ImageNet dataset [33] to extract local scene features. The predicted sequence image is input in the form of a 4D array, and each dimension represents the number of observation frames, image rows, image columns, and image channels. We extract the output with size (512, 14, 14) from the fourth maximum pooling layer of VGG19, and then use the average pooling layer with a 14 × 14 kernel for pooling to obtain the 512D feature vector. Finally, the feature vectors of each frame are connected to obtain the spatio-temporal features of (n, 512), where n represents the number of observation frames. The network structure is shown in Figure 8.

#### 3.4.3. Global Context

The information in the global scene, mainly the traffic information, will have an important impact on pedestrians crossing behavior. The vehicle information on the road, including the distance to the pedestrian, vehicle speed and speed change, must be considered when pedestrians cross the street.

**Figure 9:**
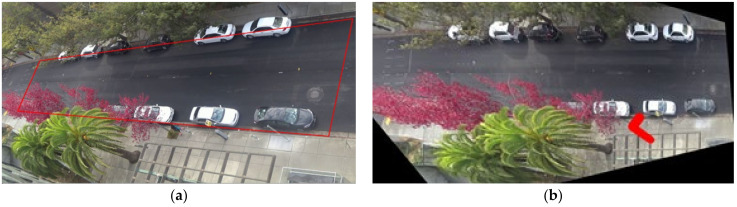
Comparison before and after image preprocessing. (**a**) is the picture before processing, (**b**) is the result of highlighting the target pedestrian and perspective transformation.

In order to highlight the target pedestrian in the image, we use two line segments with a width of 60 pixels to represent the target pedestrian. One indicates the pedestrian’s body position and the other indicates the pedestrian’s direction. This can not only connect the road context with the only target pedestrian, but also more directly judge the target pedestrian’s understanding of the current road environment through the pedestrian head direction, such as whether the pedestrian pays attention to the approaching vehicle. At the same time, since the surveillance video camera has an unchanged viewing angle, we perform a fixed perspective transformation on the input image. In this way, the near end and the far end of the camera can be at the same scale, avoiding the problem that the size and speed of the target at the far end of the camera are too small due to the viewing angle. Figure 5a shows four points used to calculate the perspective transformation matrix, which are manually marked. The coordinates of the corresponding four points after perspective transformation are set as (200, 200), (200, 680), (1870, 200), (1870, 680), respectively. The images before and after transformation are shown in the Figure 9.

**Figure 10:**
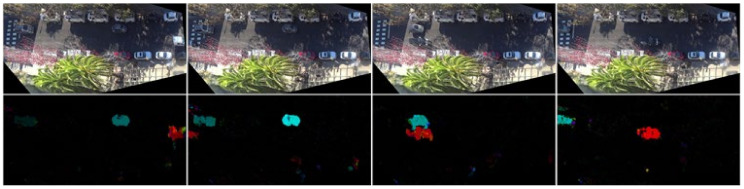
The result of optical flow method preprocessing picture. The upper picture is the road scene at different time points, and the lower picture corresponds to the optical flow field calculated by the upper picture and its previous frame.

In addition, in order to focus on the moving vehicles on the road, we use the dense optical flow method to obtain the optical flow field of the picture. Since the road background is basically static in the scene under surveillance video, the information of moving targets can be easily extracted by optical flow method. The processing results are shown in the Figure 10. Then, the original road image and optical flow field image are transformed to (224, 224), which are, respectively, input into the convolution neural network to extract the original global features and motion features. The network used is the same as the extraction of local environment features. Finally, the feature vectors of each frame are connected to obtain two final features of (n, 512), and n represents the number of observation frames.

## 4. Experiment and Results

### 4.1. Benchmark and Metrics

According to the benchmark proposed in [5], the following indicators are used to evaluate the test results: accuracy, F1 parameter, precision, recall rate and area under the curve (AUC).

Accuracy represents the proportion of correct data predicted. Precision represents the correct proportion of those data whose prediction is positive. Recall rate indicates the correct proportion predicted by positive samples. Their calculation formula is shown in Equations (7)–(9).

(7) $$\mathrm{accuracy}=\frac{TP+TN}{TP+FN+FP+TN}$$

(8) $$\mathrm{precision}=\frac{TP}{TP+FP}$$

(9) $$\mathrm{recall}=\frac{TP}{TP+FN}$$

where: *TP* represents the number of samples with positive label and positive prediction result. *TN* represents the number of samples with negative label and negative prediction result. *FP* represents the number of samples with negative label and positive prediction result. *FN* represents the number of samples with positive label and negative prediction result.

Ideally, the higher precision and recall, the better, but the actual situation is that the two affect each other: the pursuit of high accuracy rate will lead to low recall rate; the pursuit of high recall rate will usually reduce the accuracy rate. In order to balance the accuracy and recall rates, the F1 parameter is introduced, and its calculation formula is shown in Equation (10).

(10) $$F1=\frac{2*\mathrm{precision}*\mathrm{recall}}{\mathrm{precision}+\mathrm{recall}}$$

In the case of binary event anticipation, AUC reflects the balanced accuracy of the algorithms.

(11) $$\mathrm{AUC}=\frac{\sum I\left(P_{p},P_{n}\right)}{M*N}$$

where *M* is the number of positive samples, N is the number of negative samples, $P_{p}$ is a score of positive samples, $P_{n}$ is a score of negative samples, $I\left(P_{p},P_{n}\right)$ = 0($P_{p}<P_{n}$), 0.5($P_{p}=P_{n}$) or 1($P_{p}>P_{n}$).

We compare the proposed algorithm with the following four benchmarks to evaluate the performance of our algorithm.

Single RNN [34]. First, all input features are connected into a vector. Then it is input into the recurrent neural network for recursion. Finally, the full connection layer is used for prediction.

Multi RNN [35]. Each input is input into the recurrent neural network, and then the hidden features of each RNN output are connected into a vector. Finally, the full connection layer is used for prediction.

SF RNN [11], a stacked RNN network. Different features are processed in layers and gradually fused at each layer. The more complex features are fused at the bottom, and the simpler features are fused at the top.

PCPA [13]. The attention module is used. After GRU calculation for each input, the attention module is used for time attention. The attention module is applied to the branch output again to realize modal attention after connecting the output results.

### 4.2. Quantitative Experiment

**Figure 11:**
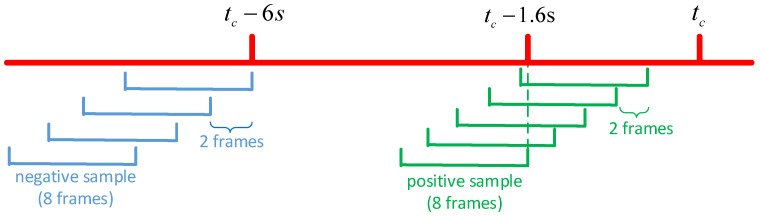
Schematic diagram of experimental sample extraction. Where $t_{c}$ represents the time when pedestrians begin to cross the street. The red line indicates the time line of pedestrian crossing behavior. The green and blue lines represent the extracted positive and negative samples, respectively.

According to the summary in [13], in the current research on the prediction of pedestrian crossing behavior, most of the observation time is about 0.5 s. The TTP is mostly in the range of 1 s to 2 s. The experimental data of some studies are taken from the whole process of crossing the street, while others are taken from the part before crossing the street. Since the prediction of the time when pedestrians begin to cross the street is the focus and difficulty in the research. Therefore, we tested each model under 8 frames (0.53 s) observation time and 24 frames (1.6 s) time to prediction (TTP). The video clips are divided into training set and test set in the ratio of 3:1. Due to the limitation of our data volume, we will extract five positive sample sequences from a sample of crossing pedestrian. For each pedestrian crossing the street, take [$t_{c}$−31, $t_{c}$−16], a total of 16 frames of data. $t_{c}$ is the number of frames at the beginning of pedestrian crossing behavior. Continuously taking sequences with a length of 8 frames as positive samples at an interval of 2 frames, and finally a total of 5 sequences of [$t_{c}$−31,$\;t_{c}$−24], [$t_{c}$−29,$\;t_{c}$−22], [$t_{c}$−27,$\;t_{c}$−20], [$t_{c}$−24,$\;t_{c}$−18] and [$t_{c}$−22,$\;t_{c}$−16] can be obtained. The negative sample of the model is the sequence of more than 6 s before $t_{c}$, which are also collected at an interval of 2 frames. The experimental sample extraction process is shown in Figure 11. Finally, the data volume of the dataset is doubled by horizontal mirroring. The final training set is about 3000 samples and the test set is about 1000 samples, of which the proportion of positive and negative samples is about 1:1.

The experimental results are shown in Table 2. It can be seen from the results that the methods proposed in this paper are optimal with respect to all metrics except the recall rate. Although the recall rate of PCPA model is the highest, its accuracy and precision are 3% and 5% lower than our method. This shows that our model is more sufficient for the fusion of multi-source inputs. Since our model does not use RNN alone for prediction of each input, such as PCPA and multi RNN, but combines two complex input features in a cross stacking way. It also does not make simple connection and fusion such as single RNN, because there are differences in the dimensions of different features. In addition, this stacking method enables local and global contex features to have two levels of recursion (1 layer of RNN and 2 layers of RNN), which can also make the recursion of features more sufficient.

In Table 3, the run time performance of our proposed framework in comparison to the other approaches is listed. Since all models use the same input, we test the acquisition of input and the calculation time of prediction model separately. It can be seen from the table that although our model is not excellent in time, the main time-consuming of the algorithm comes from the acquisition of model input, that is, the extraction of local and global context features. Therefore, the time-consuming prediction model does not need too much attention. All the run-time analysis experiments run on the same PC with an Intel i7 CPU and an Nvidia GTX1080Ti.

### 4.3. Effect of Pedestrian Speed

We counted the number of crossing samples and not crossing samples correctly predicted by all models, some models or no models. Correspondingly, the samples are divided into simple samples, medium samples and difficult samples. At the same time, we divide pedestrians into three categories according to their moving speed: fast moving, medium moving and slow moving. The relationship between pedestrian moving speed and sample difficulty level is studied. The moving speed of pedestrians is calculated according to Equation (12) where, n is the length of observation sequence, $lx^{i}$ and $ly^{i}$ are the horizontal and vertical coordinates of pedestrian waist center in the i-th frame of the sequence, respectively. Since the interval time of each frame is the same, the speed is not divided by time.

(12) $$v=\overset{n}{\underset{i=2}{\sum }}{\left(lx^{i}-lx^{i-1}\right)}^{2}+{\left(ly^{i}-ly^{i-1}\right)}^{2}$$

**Figure 12:**
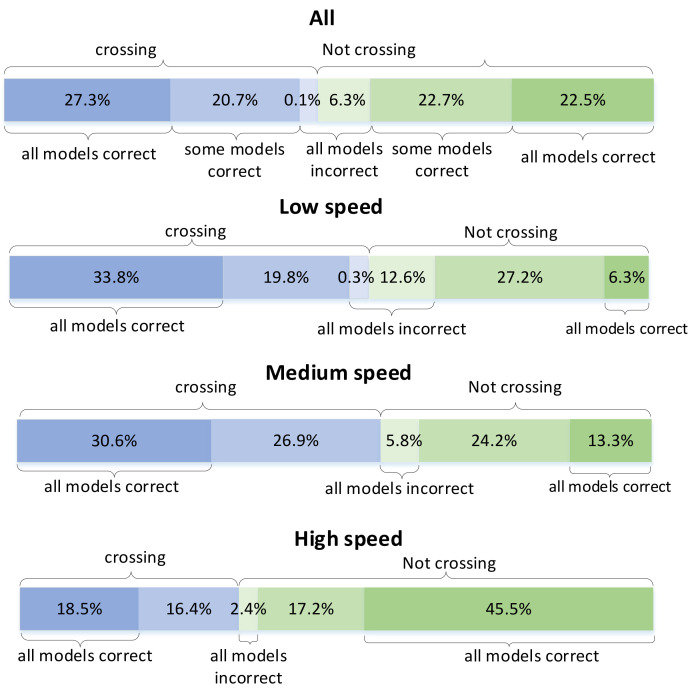
Proportion of crossing and not crossing samples in low-speed, medium-speed and high-speed pedestrians that are correctly classified by all/some/none models.

The proportion of pedestrian samples at the three speed categories after classification is about 1:1:1. Then, we count the number of simple, medium and difficult pedestrian samples at different speeds. The results are shown in the Figure 12. As can be seen from the figure, for the low-speed pedestrian sample, the simple sample accounted for 39.7% and the difficult sample reached 12.9%. For the sample of medium speed pedestrians, the simple sample accounted for 43.9% and the difficult sample accounted for 5.8%. For the high-speed pedestrian sample, the simple sample accounts for 64%, and the difficult sample is only 2.4%. The slower the pedestrian speed, the more difficult the model is to predict the pedestrian, which is particularly obvious in the negative sample. The reason may be that our positive and negative samples are from pedestrians who are about to cross the street. Slow negative samples are often pedestrians who slow down or stop on the street to observe the road conditions. If the current road context is complex, pedestrians will wait at the roadside for a long time. The prediction will be wrong if the model does not fully understand the current road context.

### 4.4. Effect of Time to Prediction

In order to explore the performance of the model under different TTP we carried out further experiments by changing the TTP. We fixed the observation length to 8 frames (about 0.53 s), increased the TTP from 0.4 s to 2 s, and the step size was 0.2 s. In each group of experiments, the last frame of the positive sample observation sequence starts from t frame and takes 5 consecutive sequences at intervals of 2 frames, that is, the end frames of each observation sequence are t, t + 2, t + 4, t + 6 and t + 8, respectively. $t=t_{c}-t_{p}$, where $t_{c}$ is the number of frames at the beginning of pedestrian crossing behavior, and $t_{p}$ is the number of frames corresponding to the TTP. Since the dataset does not label the poses of pedestrians who have not crossed the street, we choose the sequence whose end frame is more than 6 s before the pedestrian crossing behavior as the negative samples. We divide the video into training set and test set in the ratio of 3:1. Finally, the total sample data of each group is about 4000, and the proportion of positive and negative samples is about 1:1.

**Figure 13:**
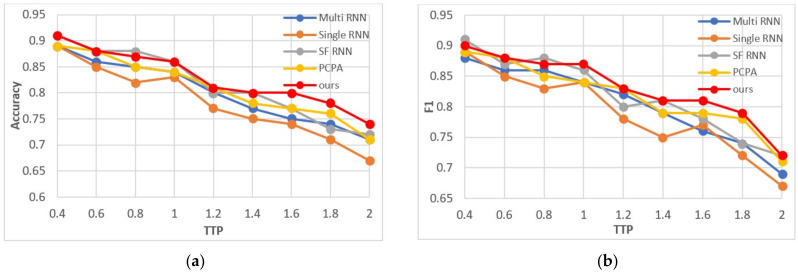
When the observation time is 0.53 s, the TTP is increased from 0.2 s to 2 s to test the performance of the algorithm. (**a**,**b**) represent accuracy and F1 parameter, respectively.

According to the experimental results, we selected two indicators of accuracy and F1 parameters to show the prediction performance. As shown in Figure 13, the accuracy and F1 parameters of our model are the optimal values in most cases. At the same time, when the TTP is very short, the variation of accuracy between the models is small, because the pedestrian intention is more obvious. With the increase of TTP, the performance of all algorithms decreases gradually, but the decline speed of different models is different, and the gap of prediction results of different models also increases gradually.

### 4.5. Qualitative Experiment

**Figure 14:**
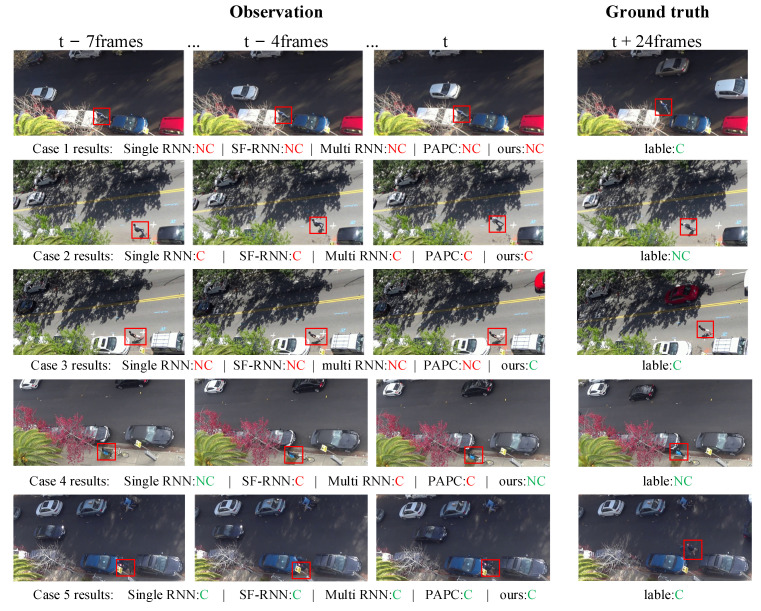
Examples of qualitative experimental results of pedestrian crossing behavior prediction. In each group of experiments, the left three pictures represent the observation frames of frames 1, 4 and 8, respectively, and the rightmost picture represents the real picture at the predicted time point. Where, C represents crossing the street, NC represents not crossing the street, red indicates wrong prediction, and green indicates correct prediction.

We also conducted some case studies to analyze the behavior types of the pedestrians’ crossing. The cases are displayed in Figure 14.

In case 1, the pedestrian will cross but the prediction results of all models are wrong. The reason may be that the pedestrian waited too long on the street, the current situation of road vehicles is complex, and the road environment changes rapidly. This makes the model unable to accurately predict the behavior over a long period of time. In case 2, the pedestrian will not cross but all model’s predictions are wrong. It can be seen from the observation sequence pictures that this is caused by the sudden change of pedestrian’s intention and trajectory. This is also a situation that cannot be accurately predicted by models or even human drivers. In case 3, the pedestrian will cross the street. The prediction of our model is correct, but the other models are wrong. It can be seen from the picture sequence that the pedestrian stayed on the roadside for a long time due to the complex road environment, but he shows behaviors of leg lifting which shows that he is eager to cross the street. Before the vehicle has completely passed, pedestrians cross the street obliquely in the vertical direction, which is a common way for pedestrians to cross the street when they encounter passing vehicles in their daily life. In case 4, the target pedestrian walked towards the road, but then his speed suddenly slowed down, resulting in some model prediction errors. In case 5, the pedestrians will cross the street, and all models predict it correctly. At that time, the target pedestrian was already standing next to the road and was ready to cross the street. The companion next to him was also preparing to cross the street. The pedestrian’s movement was coherent, so all models could predict it correctly.

## 5. Discussion

This paper presents a pedestrian crossing behavior prediction model based on surveillance video. Compared with traditional vehicle-based video, surveillance video can capture richer road and vehicle information, which will have a large impact on pedestrian cross-street behavior. In addition, we propose a new feature fusion method, which improves the prediction performance of the model, and obtains higher accuracy, F1 parameters, etc. than the baseline method. When TTP is less than 1.6 s, the accuracy and F1 score of the model can reach more than 80%. This study can be used in the assistant system of auto-driving to warn pedestrian crossing behavior through edge calculation, so as to enhance the safety performance of auto-driving.

However, due to the limited ability and energy, some aspects of the algorithm need to be further improved:

(1) The surveillance video does not capture all the information on the road, especially the information on the right side of the camera. Pedestrians can observe farther road information than cameras. Therefore, many vehicles that affect pedestrian crossing behavior do not appear in the video.
(2) The rules of pedestrian-vehicle interaction when pedestrians cross the street are complex and changeable. The amount of data in the current dataset is difficult to make the model fully learn these rules. Our positive and negative samples are from different stages of pedestrians who will cross. There is a lack of samples of pedestrians who won’t cross. This makes the model less robust and reduces a certain accuracy. These reasons lead to the rapid reduction of model accuracy with the increase of TTP.
(3) The proposed method relies on the labeling of key points to encode human posture, which restricts the practical use of the proposed method.

Future research will start from these aspects, consider using multiple cameras to broaden the observation field of vision, and increase the number and integrity of datasets to improve the robustness and accuracy of the model. In addition, we will study the detection of human posture key points in surveillance video to realize an end-to-end pedestrian crossing behavior prediction model. At the same time, we also believe that the representation of pedestrian posture is not necessarily the key points of posture. In the future, we will also try different inputs that can contain pedestrian posture information.

## 6. Conclusions

This paper focuses on pedestrian crossing behavior prediction based on surveillance video. A new spatio-temporal feature fusion network based on stacked GRU is proposed. The algorithm predicts the pedestrian crossing behavior by fusing the features of pedestrian posture, local context and global context. Quantitative and qualitative experiments are carried out using the pedestrian crossing behavior prediction dataset under surveillance video. The results show that our method has the best performance compared with other baseline methods. Then we counted the proportions of simple, medium, and difficult samples in pedestrian samples with different speeds. The results show that the slower the pedestrian movement, the more difficult the sample prediction. We also demonstrated the performance of each model at different prediction times. Experiments show that when the prediction time is short, the accuracy of each model is close. With the increase of prediction time, the performance of all models decreases. However, the performance gap between the models gradually widens with different decline speeds. The research of this paper proves the feasibility of pedestrian crossing behavior prediction based on surveillance video. It can provide a reference for the application of edge computing in the safety guarantee of automatic driving.

## Figures and Tables

**Table 1:** Data time summary.

Time	Numbers of Videos
9:00–11:00	17
11:00–13:00	40
13:00–15:00	108
15:00–17:00	104
17:00–19:00	31
Total	300

**Table 2:** The prediction results of each model on pedestrian crossing behavior under 8 frames (0.53 s) observation time and 24 frames (1.6 s) TTP. The evaluation index of the algorithm consists of accuracy, area under curve (AUC), F1 parameter, accuracy, recall and average value.

Models	Acc	AUC	F1	Prec	Recall	Average
Single RNN	0.74	0.74	0.77	0.67	0.88	0.760
Multi RNN	0.75	0.75	0.76	0.70	0.84	0.760
SF RNN	0.77	0.77	0.78	0.73	0.82	0.774
PCPA	0.77	0..78	0.79	0.70	**0.90**	0.788
ours	**0.80**	**0.80**	**0.81**	**0.75**	0.88	**0.808**

The **bold** result means the best in the models. Acronyms: Acc (Accuracy), AUC (Area under the ROC Curve), F1 (F1 score), Prec (Precision).

**Table 3:** Comparison of calculation time of different methods.

Approach	Time
Single RNN	0.4 ms
Multi RNN	0.6 ms
SF RNN	0.8 ms
PCPA	0.7 ms
ours	0.7 ms
Extraction of local context	7.6 ms
Extraction of global context	17.4 ms

## Data Availability

The data is based on BDD data and we have no authority to disclose it.
